# Phytocannabinoids and Male Fertility: Implications of *Cannabis sativa* and the Endocannabinoid System in Reproductive Regulation

**DOI:** 10.3390/plants15030473

**Published:** 2026-02-03

**Authors:** Ochuko L. Erukainure, Jennifer Nambooze, Chika I. Chukwuma

**Affiliations:** 1 Department of Microbiology, School of Life Sciences, University of KwaZulu-Natal, Durban 4000, South Africa; 2 Department of Biochemistry, School of Life Sciences, University of KwaZulu-Natal, Durban 4000, South Africa; 3 Department of Chemistry, Faculty of Natural and Agriculture Science, University of Free State, Bloemfontein 9300, South Africa; n.jennifer33@gmail.com; 4 Centre for Quality of Health and Living (CQHL), Faculty of Health and Environmental Sciences, Central University of Technology, Bloemfontein 9301, South Africa; cchukwuma@cut.ac.za

**Keywords:** cannabidiol, cannabis, endocannabinoid system, male infertility, spermatogenesis, and Δ9-tetrahydrocannabinol

## Abstract

*Cannabis sativa*, a species within the Cannabaceae family, produces a diverse range of phytochemicals, notably cannabinoids and terpenoids, with significant physiological and pharmacological relevance. Among its phytochemicals, Δ9-tetrahydrocannabinol (THC) and cannabidiol (CBD) are the most studied for their psychoactive and medicinal properties. However, emerging evidence indicates that chronic or excessive exposure to these phytocannabinoids may adversely affect male fertility. This review synthesizes current knowledge on the influence of C. sativa and its constituents on the male reproductive system, with emphasis on spermatogenesis, sperm function, hormonal regulation, and the role of the endocannabinoid system (ECS). Experimental and clinical studies demonstrate that cannabinoids interact with CB1 and CB2 receptors expressed in the testes, epididymis, and spermatozoa, thereby modulating testosterone synthesis, sperm motility, morphology, and capacitation. THC, in particular, disrupts the hypothalamic–pituitary–gonadal (HPG) axis, leading to reduced luteinizing hormone and testosterone levels, impaired mitochondrial activity, and abnormal sperm morphology. Although CBD exhibits anti-inflammatory and antioxidant properties, its long-term impact on reproductive function remains uncertain. The review further highlights the complex interplay between exogenous cannabinoids and the endogenous ECS in maintaining reproductive homeostasis. Understanding these molecular mechanisms is critical for balancing the therapeutic potential of Cannabis-derived products with their reproductive risks. This knowledge could inform safe medicinal applications and contribute to the development of targeted cannabinoid-based therapies for male infertility.

## 1. Introduction

The genus *Cannabis* is part of the Cannabaceae family, and includes three species, *C. sativa*, *C. indica*, and *C. ruderalis*, with the former being the most studied species. *Cannabis sativa*, sometimes referred to as weed, marijuana, or pot, is indigenous to South and Central Asia, and has been utilized for ages in a variety of traditional medical treatments as well as for spiritual and leisure uses [1]. Cannabis products mainly come from the female *Cannabis sativa* plant, with the primary psychoactive ingredient being delta-9-tetrahydrocannabinol (THC) [2]. The highest concentrations of THC are found in the plant’s flowering tops, with lower amounts in the leaves, stems, and seeds [3,4,5].

Cannabis is commonly consumed by smoking, usually via a rolled “joint” similar in size to cigarettes, with smokers often holding their breath and taking deep inhalations to enhance THC absorption in the lungs [6,7], thereby conveniently delivering the desired euphoric effects. An average joint contains about 0.5 to 1 g of cannabis (20% to 70% THC), with 5–24% of THC being absorbed by the body [2]. For infrequent users, as little as 2–3 mg of bioavailable THC can produce a “high” effect, whereas frequent users develop tolerance and may consume five or more joints to achieve a similar effect [8,9].

Cannabis can cause a range of physical and mental effects, including euphoria, a change in perception of duration, difficulty focusing, memory retention, poor balance and fine motor skills, calmness, and increased appetite [10]. When smoked, these effects appear within minutes, but when consumed orally, they may take up to 90 min due to the digestion and absorption process. Depending on the dose, the effects can last two to six hours. At high doses, cannabis may cause anxiety, delusions, paranoia, hallucinations, panic, and even psychosis. There is a strong correlation between cannabis use and an increased risk of psychosis, though causality remains debated. Children of mothers who use cannabis during pregnancy may face physical effects like increased heart rate, respiratory problems, nausea, and behavioral issues; short-term effects may also include red eyes and dry mouth [2]. Long-term effects can include chronic cough, susceptibility to respiratory infections, addiction, reduced cognitive ability in individuals who began regular use as teenagers, and cannabis hyperemesis syndrome [11].

Cannabis is also utilized for therapeutic purposes, with CBD being the most studied phytocannabinoid for its potential to treat conditions like anxiety, psychosis, and epilepsy, and to reduce pain [12,13]. Cannabis has been explored in the management of various cancers, including glioblastoma multiforme, non-small cell lung cancer, renal cell carcinoma, and breast cancer [14,15,16]. Despite its pharmacological properties, there are controversies on the effect of cannabis and its phytocannabinoids on male fertility. Some studies indicated the therapeutic effect of cannabis on male fertility [17,18]. Others indicate associated chronic or excessive exposure to cannabis and its phytocannabinoids with adverse effects on male fertility [18,19,20,21]. Thus, this review is aimed at synthesizing current knowledge on the effect of *C. sativa* and its constituents on the male reproductive system, with emphasis on spermatogenesis, sperm function, hormonal regulation, and the role of the endocannabinoid system (ECS).

## 2. Materials and Methods

Relevant literature was identified and carefully chosen through comprehensive searches of PubMed, Scopus, and Google Scholar using the keywords *Cannabis sativa*, phytocannabinoids, endocannabinoid system, and male fertility, with emphasis on studies and reviews published between 2010 and 2025.

### 2.1. The Genus Cannabis

**Figure 1:**
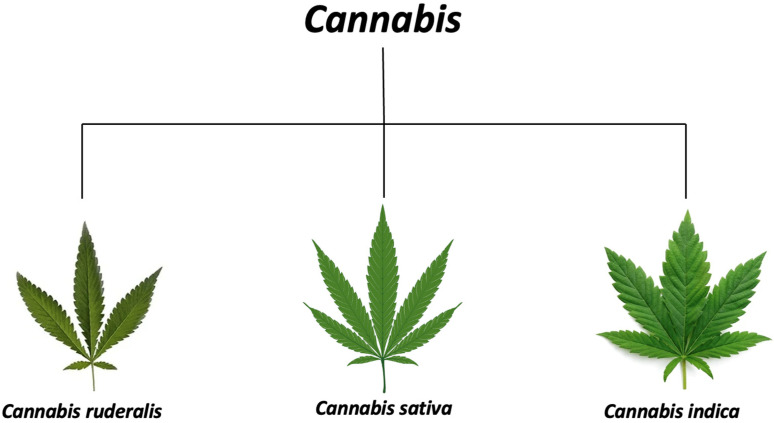
The genus *cannabis* and its species.

Cannabis is categorized within the Cannabaceae family of flowering plants, with three distinct species identified: *Cannabis ruderalis*, *Cannabis sativa*, and *Cannabis indica* (Figure 1) [22]. It is also possible for *C. ruderalis* to be a part of *C. sativa*, or for all three to be classified as subspecies of *C. sativa*, or for *C. sativa* to be considered a single, undivided species [22]. The genus is considered to be native to and originated from Asia.

### 2.2. Cannabis indica

*Cannabis indica* is an annual plant species belonging to the genus *cannabis*, native to the Hindu Kush mountains in Southern Asia. It is known for producing significant amounts of tetrahydrocannabivarin (THCV) and tetrahydrocannabinol (THC). Today, it is extensively cultivated in regions such as Thailand, China, Nepal, Afghanistan, India, Pakistan, and parts of southern and western Africa, with cultivation methods including hashish production in India. The elevated concentrations of THC and THCV result in euphoric effects, making *C. indica* famous for a variety of uses, including recreational applications, clinical research, and the exploration of potential new drugs or alternative medicine options [23].

### 2.3. Cannabis ruderalis

*Cannabis ruderalis* is a species native to Russia and Central and Eastern Europe. Unlike *C. indica* and *C. sativa*, it does not depend on photoperiod conditions to flower and contains lower levels of the psychoactive compound tetrahydrocannabinol (THC). Due to its phenotypes and distinct characteristics that differentiate it from *C. sativa* and *C. indica*, some researchers classify *C. ruderalis* as a separate species [24]. In contrast, others debate whether it should be considered a subspecies of *C. sativa*. In traditional Russian and Mongolian medicine, *C. ruderalis* has been used for a long time, especially to treat depression. It is infrequently employed for recreational purposes due to being one of the cannabis biotypes with the lowest THC content [22]. Due to their typically higher CBD levels, *C. ruderalis* may be beneficial for treating conditions like epilepsy or anxiety [22].

### 2.4. Cannabis sativa

*Cannabis sativa* is an herbaceous, flowering plant that blooms annually [23]. Although native to Eastern Asia (India and China), it is now widespread due to extensive cultivation. Throughout recorded history, *C. sativa* has been grown for its industrial fiber, seed oil, food, and medicinal uses. Additionally, it has been employed for religious, spiritual, and recreational purposes [25].

### 2.5. Phytochemical Properties of Cannabis sativa

The plant contains various chemically active compounds, including alkaloids, flavonoids, carbohydrates, terpenoids, phytocannabinoids, fatty acids and their esters, phytosterols, phenolic compounds, amines, and amides [26]. Among these, cannabinoids’ terpene phenolic compounds are the most potent and are primarily located in the trichome cavities of the female flowers. The most powerful of the more than 100 cannabinoids discovered, trans-Δ^9^-tetrahydrocannabinol (D9-THC), is primarily responsible for the plant’s intoxicating effects [27].

### 2.6. Terpenoids

Terpenoids, also known as isoprenoids, are oxygen-containing compounds derived from terpenes that serve various functions in plants, including stabilizing membranes, acting as phytohormones, and playing key roles in processes like respiration, photosynthesis, signalling, and defence mechanisms [25]. These compounds can be linear, monocyclic, or polycyclic hydrocarbons featuring a range of functional groups such as ethers, alcohols, ketones, esters, and aldehydes [25]. Research has indicated that these compounds work synergistically with *cannabis* [25].

The flowers and leaves of *C. sativa* contain over 200 terpenoids, which are responsible for the plant’s scent and may make up 10% of its trichome content [28]. The most common and highly volatile terpenoids include pinene, limonene, and myrcene. These compounds act as antifeedants for herbivores and insect repellents. A synergistic mechano-chemical defence mechanism involving a combination of various *C. sativa* terpenoids and phytocannabinoid acids protects a range of predators [29]. Environmental factors influence the generation of terpenoids. These compounds, such as phytocannabinoids, produced as part of the plant’s defence mechanisms, become more concentrated when exposed to light, which is unpleasant for the plant. However, it decreases with higher soil fertility [29].

Terpenes and terpenoids in cannabis are categorized into several distinct groups:

- Monoterpenes (C10), which include 61 compounds, consist of two isoprene units [30,31]. These volatile substances are responsible for the characteristic aroma of cannabis [31]. The main components are trans-ocimene, linalool, β-myrcene, α-pinene, and α-terpinolene. Their concentration increases in the plant during the late harvest phase [30].
- Sesquiterpenes (C15), composed of three isoprene units, include 51 compounds. These semi-volatile compounds also play a role in the distinctive odor of cannabis [30,32]. E-caryophyllene, β-caryophyllene, E-β-farnesene, and caryophyllene oxide are the main constituents, and their concentrations are at their peak during the early harvest season [31],
- Diterpenes (C20), composed of four isoprene units, include steroids, waxes, and resins. Currently, *Cannabis sativa* contains only two known diterpene compounds: phytol and neophytadiene [33].
- Triterpenes (C30), consisting of six isoprene units, are found in plants’ roots, stem and leaves, with the highest concentrations typically located in the roots. These compounds include steroids, waxes, and resins. Friedelin, the primary compound, was first identified in 1971, along with trace amounts of β-amyrin and epifriedelin [30].

### 2.7. Phenolic Compounds

Lignans, flavonoids, and stilbenoids are the three main categories of phenolic compounds present in cannabis [30,32]. Whereas stilbenoids and flavonoids are mostly found in the leaves, stalks, and inflorescences, lignans are mostly found in the roots, seeds, and fruits [32]. As the plant matures, the concentration of these phenolic compounds reduces as they combine with terpenes to form cannabinoids [33].

### 2.8. Flavonoids

Flavonoids, the largest group of phenolic compounds, are found in the plant’s leaves, flowers, seeds, and pollen. These metabolites enhance the plant’s therapeutic properties [34,35]. Among the more than 34 flavonoids extracted by Bautista et al., some key examples include luteolin, vitexin, kaempferol, isovitexin, orientin, quercetin, apigenin, and three flavones with geranyl or prenyl groups known as cannflavins A, B, and C [34,36,37]. There are notable variations in the quantities of these compounds within *Cannabis* across different species and plant parts. Environmental factors like temperature, sunlight exposure, humidity, and precipitation influence their production [36,37]. Moreover, the formation of many compounds depends on specific conditions, including both biotic and abiotic stressors.

### 2.9. Lignans

Phenolic amides and lignanamides are the two primary types of lignans found in *C. sativa* [30]. The first group includes five compounds: N-trans-caffeoyltyramine, N-trans-coumaroyltyramine, N-trans-caffeoyloctopamine, N-trans-feruloyltyramine, and N-trans-coumaroyloctopamine, which were detected in trace amounts in cannabis, as reported by Isidore and colleagues [30].

### 2.10. Stilbenes

Stilbenes are found in the plant’s inflorescences, trichomes, leaves, and stalk. They are mainly categorized into three groups: dihydrophenanthrenes, or phenanthrenes, including seven compounds; dihydrostilbenes, encompassing twelve compounds and one canniprene, which has shown anti-inflammatory activity; and spiro-Indians, composed of sixteen compounds, including the primary two being cannabispirenone A and cannabispirone, which have been demonstrated to have anti-inflammatory and anti-cancer properties [30,36,38].

### 2.11. Phytocannabinoids

Phytocannabinoids, also known as cannabinoids, are a type of meroterpenoid characterized by a resorcinol core, typically featuring a para-positioned isoprenyl, alkyl, or aralkyl side chain [39]. The alkyl side chain often contains an odd number of carbon atoms: olivetoids have five, varinoids have three, and orcinoids have one. Phytocannabinoids are bioactive natural compounds in fungi, liverworts and flowering plants [39] and were first extracted and isolated from *C. sativa* [39]. The mammalian brain contains receptors that respond to phytocannabinoids. These receptors, known as cannabinoid receptors (CBx), are integral to the endocannabinoid system. Research in humans and animals has illustrated that the endocannabinoid system controls a wide array of biological processes, like mood, memory, drug addiction, brain reward mechanisms, and metabolic functions such as glucose metabolism, lipolysis, and energy balance [39,40].

**Figure 2:**
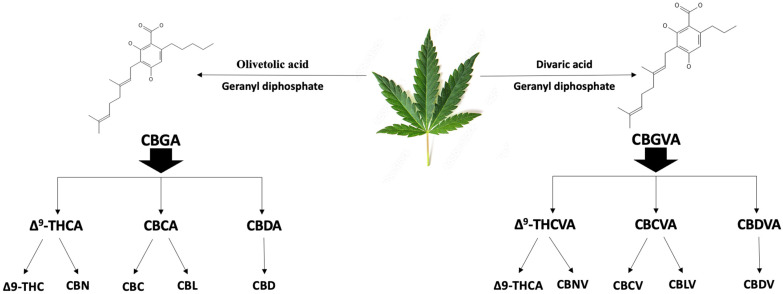
Biosynthesis of phytocannabinoids.

Currently, 538 naturally occurring phytochemicals have been identified in *C. sativa*, with over 100 of these compounds classified as phytocannabinoids due to their structural similarities [41]. These phytocannabinoids contain alkylresorcinol and monoterpene groups within their lipid structures, and are primarily found in the resinous substance that female plants’ trichomes exude [41,42]. In contrast, male *C. sativa* plants have fewer glandular trichomes, capable of producing only trace amounts of psychotropic compounds. Phytocannabinoids are characterized into two groups: neutral cannabinoids, which lack a carboxyl group, and cannabinoid acids, which contain one [43]. They further reported that *C. sativa* produces and accumulates cannabinoids in the form of cannabinoid acids, which are later converted into their neutral forms through decarboxylation [41]. As shown in Figure 2, cannabigerolic acid (CBGA) is synthesized when an aromatic prenyltransferase catalyzes the alkylation of olivetolic acid with geranyl pyrophosphate [43]. CBGA then gives rise to Δ-9-tetrahydrocannabinolic acid (Δ^9^-THCA), cannabichromenic acid (CBCA), and cannabidiolic acid (CBDA), through the action of cannabinoid synthase enzymes [44]. These compounds further undergo decarboxylation to form Δ-9-tetrahydrocannabinol (Δ^9^-THC), its chemical derivative cannabicyclol (CBL), cannabidiol (CBD), cannabinol (CBN), and cannabichromene (CBC). In contrast, cannabigerovarinic acid (CBGVA) is synthesized from geranyl diphosphate and divaric acid, giving rise to the Δ9-tetrahydrocannabivarinic acid (Δ^9^-THCVA), cannabichromevarinic acid (CBCVA), and cannabidivarinic acid (CBDVA) [45,46]. These compounds also undergo decarboxylation to yield Δ^9^-tetrahydrocannabivarinic acid (Δ^9^-THCVA), cannabicyclovarin (CBLV), cannabidivarin (CBDV), cannabinovarin (CBNV), and cannabichromevarin (CBCV) (Figure 2).

### 2.12. Subclasses of Phytocannabinoids

Phytocannabinoids are classified into ten subclasses as described below.

**Figure 3:**
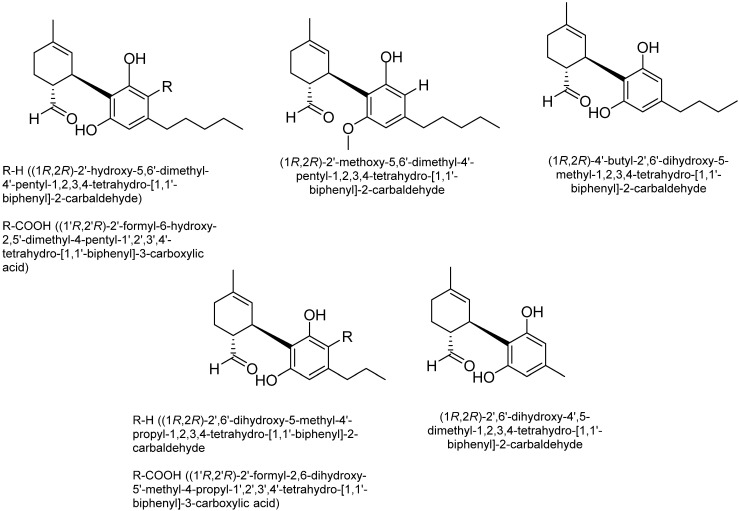
Some of the structures of cannabidiol (CBD) type cannabinoids.

Cannabidiol (CBD): The two most common phytocannabinoids found in *Cannabis* species are cannabidiolic acid (CBDA) and CBD [41]. Although CBD shares structural similarities with Δ^9^-THC, it is considered an allosteric negative modulator of CB1 and CB2 receptors. Additionally, CBD exhibits weak agonistic activity at cannabinoid receptors [47]. However, CBD can undergo electrophilic cyclization under acidic conditions, converting into Δ^9^-THC. This could also explain the formation of Δ^9^-THC in tobacco cigarettes (which contain acidic water suspensions) when CBD is introduced. Figure 3 shows some structures of CBD-type cannabinoids.

**Figure 4:**
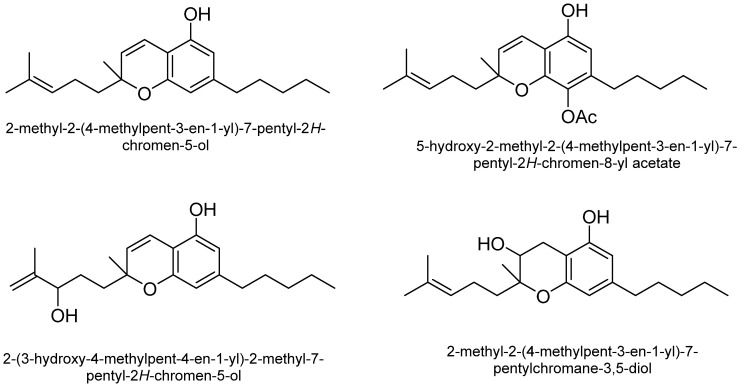
Some of the structures of cannabichromene (CBC) type cannabinoids.

Cannabichromene (CBC): CBC is among the most structurally stable phytocannabinoids [48]. The concentration of CBC is directly associated with Δ^9^-THC levels, suggesting a chemical link between the oxidase enzyme responsible for producing CBC and the conversion of CBG into Δ^9^-THC [49]. Additionally, higher levels of CBC are found during the vegetative stage of *C. sativa* compared to the reproductive stage. Additionally, while CBC is a potent activator of the transient receptor potential channel TRPA1 in inflammatory processes, CBC-based compounds do not produce CB1-mediated psychoactive effects [50]. Figure 4 shows some of the structures of CBC-type cannabinoids.

**Figure 5:**
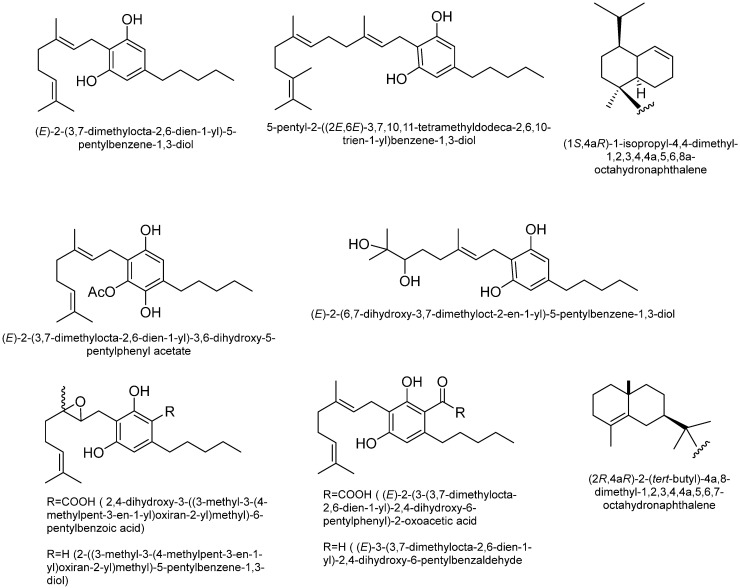
Structure of cannabigerol (CBG) type cannabinoids.

Cannabigerol (CBG): According to Izzo et al. [49], these phytocannabinoids do not produce psychotropic effects through CB1 receptor activation and have a diverse molecular structure. CBG-based compounds bind to the menthol receptor, TRPM8, inhibiting its activity in sensory neurons, and show weak inhibitory effects on the serotonin receptor, 5HT1A [51]. Additionally, CBG acts as an α-2 adrenergic receptor agonist, exhibiting analgesic, sedative, and muscle-relaxing effects by inhibiting catecholamine release [51]. Figure 5 shows the structures of cannabigerol (CBG) type cannabinoids. Figure 5 shows the structures of CBG-type cannabinoids.

**Figure 6:**
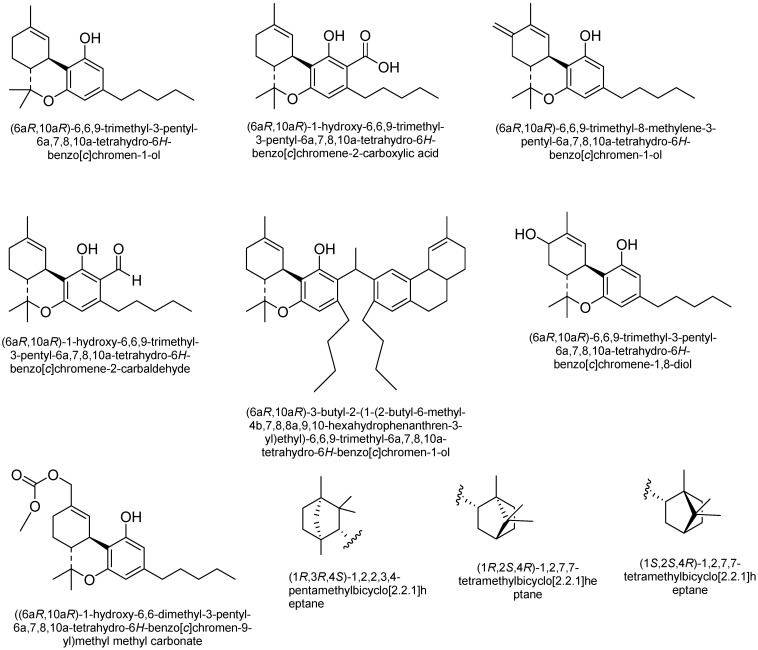
Structures of (-)-Δ^9^-trans-tetrahydrocannabinol(Δ^9^-THC) type cannabinoids.

Tetrahydrocannabinol (THC): The main psychoactive compound in *C. sativa* is trans-Δ-9-tetrahydrocannabinol (Δ^9^-THC) [52]. The primary precursors of Δ^9^-THC are acids A and B, which do not have psychoactive effects. This group also includes several stereoisomers of Δ^9^-THC, either degradation products or compounds formed through enzymatic activity. Δ^9^-THC, whether isolated and chemically purified from *C. sativa*, is unstable and typically appears as an amorphous gum that rapidly turns brown [41]. Degradation of Δ^9^-THC is estimated to occur in approximately 10% of the pure compound, mainly through oxidation; however, the principal end metabolite, CBN, is found at much lower levels. As a result, *C. sativa* has alternative metabolic pathways for the breakdown of Δ^9^-THC [23,41]. One of the most stable metabolites is Δ^8^-THC, which forms through the isomerization of Δ^9^-THC under acidic conditions, resulting in a shift of the endocyclic double bond. Trihydrocannabinol, found in cannabis pollen, can also be derived from dihydrocannabinol, a precursor of Δ^9^-THC. Other Δ^9^-THC isomers have been identified, including Δ^6a^- and Δ^10a^-THC, which are not naturally occurring but are produced via oxidative aromatization of Δ^9^-THC, and cis-Δ^9^-THC, which may be a chemical artifact [41]. Δ^9^-THC is associated with psychoactive side effects, including anxiety, paranoia, altered perception, and cognitive impairments. The structures of Δ9-THC-type cannabinoids are presented in Figure 6.

**Figure 7:**
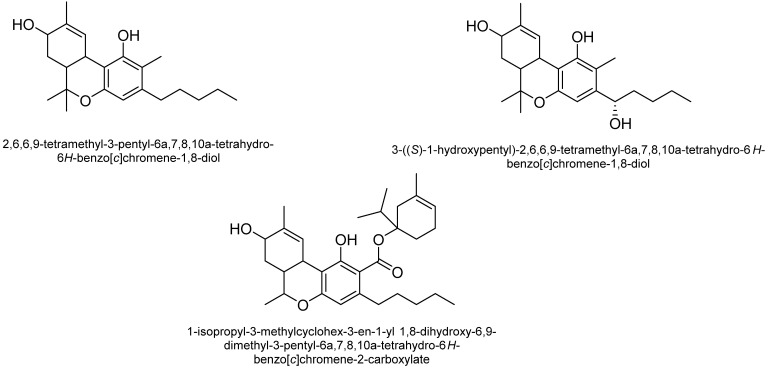
Structures of cannabinol (CBN) type cannabinoid.

Cannabinol (CBN): The amount of CBN in *C. sativa* is influenced by factors such as age and storage conditions. CBN is notably stable when it comes to oxidative degradation. Additionally, the spontaneous oxidative aromatization of Δ9-THC in *C. sativa* produces compounds similar to those resulting from CBN breakdown. Figure 7 shows some of the structures of CBN-type cannabinoids.

Δ-9-tetrahydrocannabivarin (D9-THCV): Another THC-related compound, Δ-9-tetrahydrocannabivarin (D9-THCV), is predominantly found in Pakistani *C. sativa* hashish [53]. D9-THCV is a CB1 receptor antagonist, and it inhibits the effects of Δ^9^-THC on food intake in mice at low doses (<3 mg/kg) [54]. It can also activate CB2 receptors at higher doses (10 mg/kg) [55].

**Figure 8:**
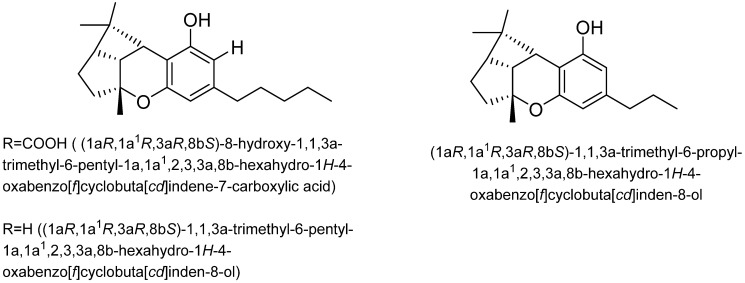
Structures of cannabicyclol (CBL)-type cannabinoids.

Cannabicyclol (CBL): Although its biological properties are not yet fully understood, it is formed by the oxidation of CBC in a process that can be triggered by heat, age, and UV light [41]. Some structures of CBL-type cannabinoids are shown in Figure 8.

**Figure 9:**
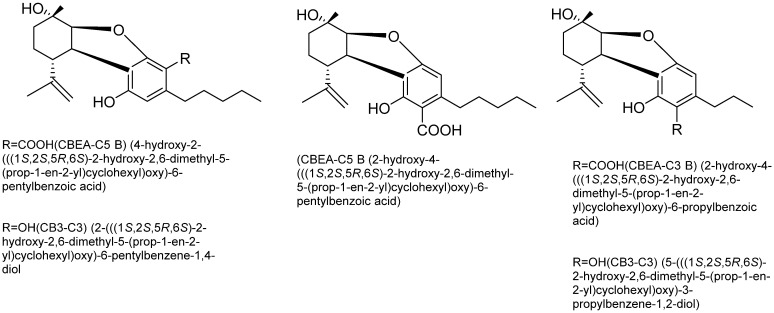
Structures of CBE-type cannabinoids.

Cannabielsoin (CBE) Type: Cannabielsoic acid B (CBEA-C5 B), cannabielsoic acid B-C3 (CBEA-C3 B), and C3-cannabielsoin (CB3-C3) are three of the five known cannabielsoin (CBE)-type cannabinoids, all sharing the same absolute configuration (5aS,6S,9R,9aR) [56]. Some structures of CBL-type cannabinoids are shown in Figure 9.

**Figure 10:**
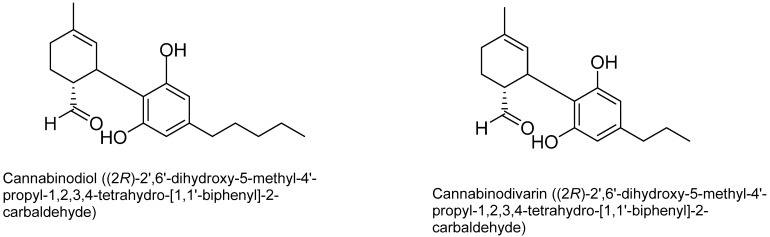
Structures of cannabinodiol (CBND) type cannabinoids.

Cannabidiol (CBND): The aromatized cannabidiol derivatives (CBD) are classified as cannabidiol-type cannabinoids. To date, the only two compounds from this subclass identified in *C. sativa* are cannabinodiol (CBND-C5) and CBND-C3 (cannabinodivarin) [57] (Figure 10).

**Figure 11:**
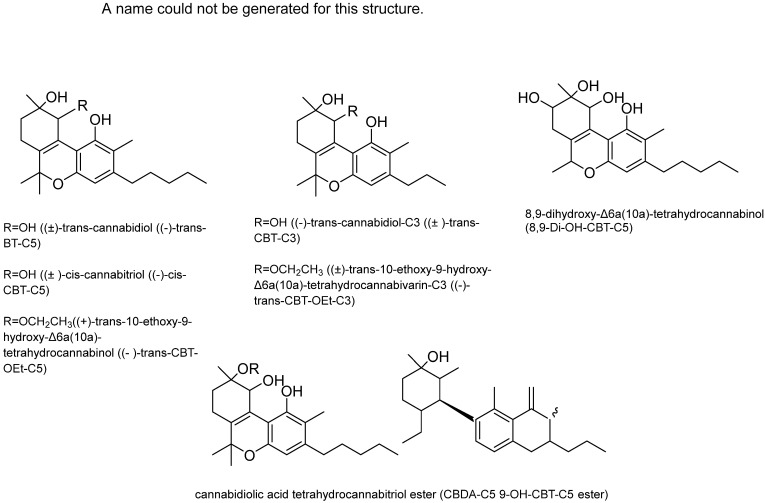
Cannabitriol (CBT) type cannabinoid structures.

Cannabitriol (CBT): In 1966, Obata and Ishikawa published the first report on cannabitriol [58]. Ten years later, the compound’s structure was elucidated [59]. An X-ray study then ascertained the chemical’s configuration [26]. Additionally, a previous review mentioned 9 CBT-type cannabinoids, including: (+)-trans-10-ethoxy-9-hydroxy-Δ6a(10a)-tetrahydrocannabinol ((-)-trans-CBT-OEt-C5), (±)-trans-cannabidiol ((-)-trans-BT-C5), (±)-trans-10-ethoxy-9-hydroxy-Δ6a(10a)-tetrahydrocannabivarin-C3 ((-)-trans-CBT-OEt-C3), (+)-trans-cannabidiol ((+)-trans-CBT-C5) (57), (-)-trans-cannabidiol-C3 ((±)-trans-CBT-C3), 8,9-dihydroxy-Δ6a(10a)-tetrahydrocannabinol (8,9-Di-OH-CBT-C5), (±)-cis-cannabitriol ((-)-cis-CBT-C5, a CBT-C3 homolog with unknown stereochemistry (CBT-C3-homologue) and cannabidiolic acid tetrahydrocannabitriol ester (CBDA-C5 9-OH-CBT-C5 ester) [26] (Figure 11). However, ethanol was employed to isolate the two ethoxy cannabitriols, (-)-trans-CBT-OEt-C3 and (-)-trans-CBT-OEt-C5, from *C. sativa* [26].

## 3. Medicinal Properties of Phytocannabinoids

Numerous *C. sativa* formulations, both natural and synthetic, are used in conjunction with treatment strategies for a range of illnesses. The endocannabinoid system (ECS) is widely distributed across the brain and peripheral areas, indicating that its activation or inhibition regulates many diseases [47]. Phytocannabinoids typically influence the regulation of ECS-related enzymes and receptors, such as G protein-coupled receptors (GPCRs) [25]. Several factors, including the method of administration, dosage, and frequency of use, influence the pharmacological effects of cannabinoids.

The ECS influences various conditions, including neuroinflammatory disorders and metabolic disorders. Specifically, cannabinoids modulate neuroinflammation and appetite regulation in the brain while also affecting peripheral metabolic responses in adipose tissue, muscles, and liver, as well as the anti-inflammatory responses of blood cells [47].

However, short-term adverse effects have been associated with *cannabis*, including anxiety, hallucinations, confusion, nausea, feeling disoriented, happiness, unconsciousness, sleepiness, vomiting, dry mouth, dizziness, asthenia, diarrhoea, balance issues, and anxiety [60,61].

### 3.1. The Endocannabinoid System

**Figure 12:**
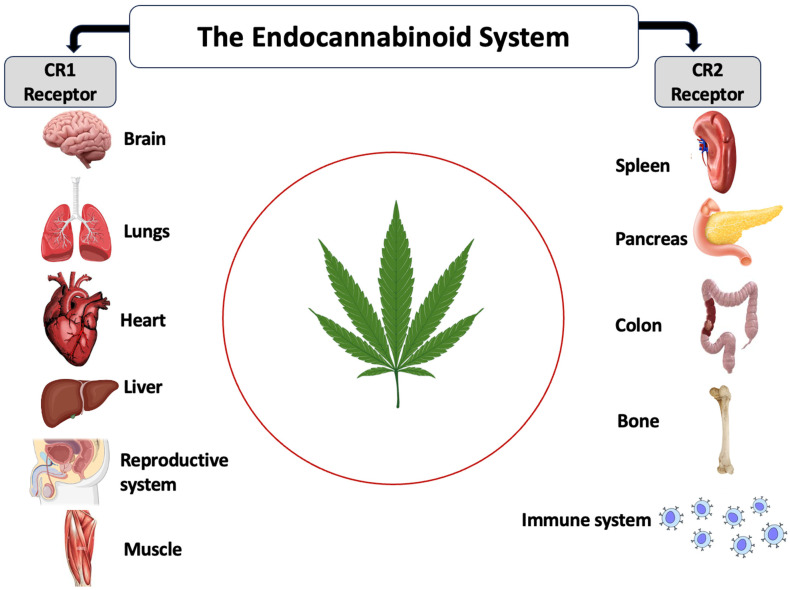
The endocannabinoid system and its receptors.

The endocannabinoid system (ECS) is a complex network of receptors, enzymes, and endocannabinoids that help regulate a variety of physiological processes and maintain homeostasis (balance) in the body [62]. It plays a key role in many functions, including mood, appetite, sleep, pain sensation, immune response, and more. Endocannabinoids are lipid-based neurotransmitters that are produced naturally by the body. Two of the most well-known endocannabinoids are Anandamide (AEA), often called the bliss molecule, which is involved in regulating mood, stress, and pleasure, and 2-Arachidonoylglycerol (2-AG), a compound that plays a role in immune function, appetite regulation, and pain perception [63]. The endocannabinoids bind to specific receptors in the body, primarily of two types (Figure 12), which include CB1 receptors, mostly found in the brain and central nervous system. They play a role in mood, memory, coordination, and appetite regulation, and CB2 receptors are primarily located in peripheral tissues, especially in the immune system. They help regulate inflammation, immune responses, and pain [63]. Enzymes are responsible for breaking down endocannabinoids once they have served their purpose. Two key enzymes involved are Fatty acid amide hydrolase (FAAH), which breaks down anandamide, and Monoacylglycerol lipase (MAGL), which breaks down 2-AG [63]. The ECS regulates various bodily functions, including pain, inflammation, mood, emotional regulation, appetite, digestion, sleep, memory, learning, and neuroprotection.

Phytocannabinoids such as THC and CBD can interact with the ECS [63]. THC is known for its psychoactive effects, as it binds to CB1 receptors in the brain. CBD, on the other hand, does not have psychoactive effects but may have therapeutic benefits, such as reducing anxiety or promoting anti-inflammatory effects.

The endocannabinoid system (ECS) is present in a wide variety of species, including fish, mussels, amphibians, birds, mammals, certain echinoderms, and even simple organisms with neural networks, such as hydra [64]. However, not all terrestrial invertebrates possess an ECS. Insects, for example, have only been shown to contain cannabinoid receptors and/or enzymes in limited studies. The use of *C. sativa* extracts as insect repellents dates to agricultural practices, as it was observed that the plant induces neurological and behavioral disruptions in insects [65].

The endocannabinoid system plays a significant neuromodulatory role in animals. Mice with specific deletion of their CB1 receptors exhibit altered pain responses, severe lack of movement, and a significantly increased mortality rate [66].

The human brain contains approximately ten times more cannabinoid receptors than opioid receptors [67]. CB1 receptors are widely expressed in regions such as the hippocampus, basal ganglia, amygdala, cortical areas, and cerebellum—all involved in mood regulation, cognition, and motor control [67]. In contrast, areas like the brainstem, which controls respiration and heart function, show lower levels of CB1 receptor expression [68]. CB1 receptors are also present in peripheral tissues such as the liver, gut, adipose tissue, and immune cells [68]. Under normal conditions, CB2 receptors are much less abundant in the brain compared to CB1 receptors. However, during inflammation, microglia and other glial cells show a marked increase in CB2 receptor expression [69]. The presence of cannabinoid receptors in the brain and the ability of cannabinoids to regulate neurotransmitters such as dopamine, glutamate, serotonin, and gamma-aminobutyric acid suggest that cannabis may have significant therapeutic potential [70]. In recent decades, various cannabinoids have been developed to modulate CB1 and CB2 receptors and influence the metabolism of endocannabinoids like AEA and 2-AG [71]. These findings underscore the important physiological role of the endocannabinoid system.

### 3.2. Involvement of the Endocannabinoid System in Male Reproductive Health

In 2019, male infertility was estimated to impact 56.5 million people globally, representing a 76.9% increase since 1990 [72]. Male infertility can be linked to low sperm count (oligospermia), poor sperm motility (asthenospermia), sperm morphology (Teratospermia), varicocele, hormonal imbalances, ejaculatory issues, genetic conditions, infections, lifestyle factors, and age [73].

In the male reproductive system, the ECS regulates sperm production, motility, and maturation. Cannabinoid receptors are present in the testes, epididymis, and sperm cells themselves, suggesting that endocannabinoids may influence sperm function [74,75,76]. Research has shown that activation of CB1 and CB2 receptors can impact testosterone production, spermatogenesis, and sperm motility. For instance, cannabinoids like tetrahydrocannabinol (THC) can reduce testosterone levels and disrupt sperm development, leading to decreased fertility in animal models. Moreover, the ECS appears to regulate the processes of sperm capacitation and acrosomal reaction, which are essential for fertilization. However, while THC and other cannabinoids can impair sperm function and fertility, the clinical relevance of these findings in humans, especially at recreational or therapeutic levels, remains under investigation.

Previous studies have mostly concentrated on investigating the reproductive toxicity associated with psychoactive constituents of *C. sativa*, primarily Δ^9^-THC [77]. As an ECS agonist, Δ^9^-THC competes with the system’s natural ligands, disrupting homeostasis and potentially leading to pathological conditions. Most studies have previously investigated the effects of marijuana on plasma testosterone levels and testicular function in users.

Consequently, studies have shown that marijuana users exhibit lower plasma testosterone levels compared to non-users [78]. Studies on animals revealed that prolonged exposure to cannabis’ psychoactive components can lead to alterations in gonadal function, including antispermatogenesis, depleted testosterone levels, and regression of male accessory organs [60,71]. In rats, both acute and chronic administration of THC reduce steroidogenic enzyme activities, resulting in decreased production of testosterone [21]. Additionally, Leydig cells in rats and mice express CB1 receptors, and research indicates that interaction with cannabinoids inhibits steroidogenesis [79]. In vertebrates, luteinizing hormone (LH) and follicle-stimulating hormone (FSH) are crucial for spermatogenesis. Research has also indicated that endocannabinoids (eCBs) influence the production of these gonadotropins, which play roles in the functioning of Leydig and Sertoli cells. Studies have shown that cannabis use in males is associated with reduced plasma LH levels, leading to a decrease in testosterone secretion [21]. While results remain inconsistent, some studies suggest that THC does not affect FSH levels [18,80]. Khoury et al. found no effect of ever cannabis use on male fecundity biomarkers, but a higher level of FSH among ever cannabis users [18]. This increase may reflect a compensatory mechanism to alterations in testicular function [18,81,82], a process regarded as hypergonadotropic hypogonadism [83,84].

However, a link between increased marijuana use and lowered FSH concentrations has been reported [85]. It has also been reported that Δ-9-tetrahydrocannabinol (Δ^9^-THC) reduces sperm motility. Another study suggests that CB1 receptors are involved in mitochondrial activity; specifically, exacerbated CB1 activity was found to lower mitochondrial membrane potential, potentially contributing to reduced sperm motility [86]. Similarly, Badawy et al. demonstrated that administering two cannabinoids, ∆^9^-THC and ∆^8^-THC, to semen caused a concentration-dependent decrease in mitochondrial respiration [87]. They concluded that ∆^9^-THC is a more potent inhibitor of mitochondrial respiration than ∆^8^-THC [87].

Additionally, cannabis use may lead to morphological changes in spermatozoa, as depicted in mice and rats intraperitoneally injected with THC and cannabinol, resulting in abnormal sperm morphology [88,89]. In a separate study of a human population in the UK, men under 30 who used cannabis within three months prior to semen collection exhibited a significantly higher incidence of abnormal sperm morphology [90].

In an in vitro study, THC was shown to inhibit the acrosome reaction in human sperm [91]. The administration of cannabis compounds has also been reported to cause testicular degeneration in experimental animal models [92], and a reduction in prostate weight and seminal vesicles [92]. THC exerts these effects by binding to the cannabinoid receptors in the testicular ECS [92].

Unlike THC, β-Caryophyllene (β-CBP) is a selective agonist of CB2-receptor and lacks the psychotic side effects associated with CB1 receptors [93]. Olopade et al. reported the ability of β-CBP to mitigate erectile dysfunction by modulating the activities of PDE5 and NOS, and improving antioxidant enzyme activities in penile tissues [94]. This was further supported by Al-Alami et al. by demonstrating the ability of β-CBP to improve sperm motility, with concomitant reduction of sperm count, while maintaining spermatogenesis and testicular morphology in rats [95].

### 3.3. The Hypothalamus-Pituitary Axis and the Endocannabinoid System

Disruption in the hypothalamic-pituitary-gonadal (HPG) axis has been implicated in its dysfunction, as the HPG axis is a key component of the ECS [96]. The HPG axis is a highly regulated endocrine system essential for reproduction and species propagation [97]. One of the key regulators of reproductive behaviour and physiological processes is gonadotropin-releasing hormone (GnRH) [98]. It is crucial in stimulating the anterior pituitary to release LH and FSH. These pituitary hormones regulate the production and release of gonadal steroid hormones, provide feedback to the anterior pituitary and hypothalamus, and play a crucial role in the regulation of gametogenesis [98].

Furthermore, experimental evidence suggests that the ECS inhibits the release of anterior pituitary hormones [99]. Shared findings have highlighted several neural systems involved in GnRH circuitry and the inhibitory effects of ECS components on GnRH release and transcription [99]. These interactions influence gonadal functions and steroidogenesis [100].

A decrease in serum LH concentration was observed when THC was administered to the third ventricle of a male rat brain, with concomitant increase in GnRH levels in the medial basal hypothalamus [101]. Activation of CB receptors in vitro in immortalized mouse hypothalamic neuronal cell lines (GT1), which express the complete endocannabinoid system, reduced GnRH’s pulsatile production. Additionally, intracerebroventricular injection of AEA into the mediobasal hypothalamus of male rats suppressed the release of GnRH [102]. Initially, studies showed that cannabis administration to anterior pituitary cells had no impact on either basal or GnRH-stimulated LH production [92]. This suggested that cannabinoids exert an upstream effect on the pituitary’s release of LH, potentially influencing reproduction by acting on the GnRH neurons in the hypothalamus. According to Hillard’s summary, some studies demonstrate the connection between GnRH neurons and the endocannabinoid system [103].

While the receptor gene was recently identified in the anterior pituitary, in particular among the lactotrophs and gonadotrophs [101], within the hypothalamus, the localization of CB1 receptors is rather low [101], even though they are mostly distributed in different parts of the brain [86]. Particularly in the hypothalamus, GnRH-releasing neurons exhibit clear endocannabinoid transmission activity of AEA and 2-AG in conjunction with FAAH [96]. Animal studies have highlighted sex-related variations that show the pituitary of males has higher amounts of CB1 receptor transcripts than that of females. In contrast, the pituitary AEA concentration is higher in females [104].

The negative relationship between endocannabinoids and GnRH levels, mediated through the hypothalamus and anterior pituitary, decreases the production of testosterone, LH, and FSH [101]. AEA, rather than 2-AG, has been reported to reduce LH and prolactin (PRL) levels in CB1 knockout mice [100], thus indicating that the hypothalamus also contains other receptors, notably TRPV1. In males, the release of GnRH from the brain regulates the production of the gonadotropins FSH and LH. The testicular negative feedback loop within the hypothalamic-pituitary system plays a crucial role in regulating and maintaining testosterone levels [105]. Elevated testosterone levels lead to a down-regulation of GnRH, ultimately reducing LH production, while LH stimulates the release of testosterone. In contrast, endocannabinoids, particularly AEA, inhibit the release of GnRH from the hypothalamus, thereby suppressing LH production by the anterior pituitary. As a result, testosterone levels decrease, reducing CB1 receptor expression and declining endocannabinoid signalling in both the pituitary and hypothalamus [105]. Endocannabinoids and cannabinoids affect the function of GnRH neurons by indirectly altering the activity of γ-aminobutyric acid (GABA)-ergic nerve fibers. GABA-ergic nerve fibers express CB1 receptors in mice, and when activated, the release of GABA is inhibited [106]. Thereby implying that the GABA receptors on GnRH neurons are not activated, preventing the release of GnRH [102]. The direct impact of endocannabinoids on gonadotropic hormone release is evidenced by cannabinoid activation of receptors in GnRH neurons through the endocannabinoid system. These findings suggest that the endocannabinoid system, hypothalamus, and anterior pituitary glands likely share a foundational interaction preserved through evolution [104,107].

### 3.4. The Role of the Endocannabinoid System in Sperm Development

Spermatogenesis is the ongoing process that produces an enormous number of sperm, which can happen seasonally or during an individual’s reproductive lifetime. The process of spermatogenesis is tightly coordinated with spermiation, and it is necessary for the ongoing generation and preservation of sperm. Similarly, the production of progenitor cells to create sperm depends on the existence of a healthy, self-renewing spermatogonial stem cell population. A carefully coordinated sequence of mitotic and meiotic divisions precedes the phase of differentiation processes that occur during spermiogenesis. This process involves the proliferation of germ cells from spermatogonia to spermatocytes and then to elongated spermatids. The exceptional maintenance is facilitated by a complex interplay between germ cells, testicular somatic cells, steroid hormones, gonadotropins, and testicular signalling pathways [74].

Germ cells from several animals exhibit CB1, CB2, and TRPV1 receptors in addition to producing AEA (Maccarrone et al., 2005). Extracted from mice aged 7, 16, and 18 days post-partum, SPG, SPC, and Sertoli cells displaying marginally weak signals in SPT contain CB2 receptors that modulate apoptosis in these cells [74]. Studies on isolated mouse germ cells found that the meiotic transition point (SPG/preleptotene-SPC differentiation) of SPG is controlled by CB2 signaling in response to 2-AG [108]. CB2 encourages SPG’s entry into meiosis and triggers the production of pre-meiotic or early meiotic genes [108]. In mice, B-type SPG’s admission to meiosis and the leptotene stage of prophase-I is marked by a global increase in H3K4me3 enrichment, which corresponds to enhanced expression of Prdm9, a methyltransferase that trimethylates H3K4 [109]. When meiosis begins, CB2 activation causes an increase in H3K4me3, indicating that CB2 activation alters histones and regulates the expression of meiotic genes [109]. 2-AG levels are also essential for meiotic entrance and SPG proliferation, which indicates a sharp drop in SPC and round SPT in the latter [110,111]. This idea is supported by the notion that post-meiotic germ cells in the testis are the source of AEA and are essential for the degradation of 2-AG [112]. Activation of CB2 leads to phosphorylation of Erk1/2 and the activation of the MAPK pathway, resulting in the activation of the MAPK system. Furthermore, CB2 stimulation has a prodifferentiative effect on isolated SPG, thereby increasing the proportion of meiotic nuclei in leptotene to zygo-pachytene spermatocytes [108].

The framework may be altered during the various stages of spermatogenesis, according to research on eCB expression in male germ cells [108]. The enzymes NAPE-PLD and FAAH were found to rise during germ cell meiosis, with AEA concentrations remaining unchanged throughout spermatogenesis from SPG to SPT [108]. Elevated levels of FAAH have been found in mice and frogs, implying that this catabolic enzyme is consistently expressed to regulate intra-testicular anandamide levels at both the early and later stages of spermatogenesis [108,109]. Additionally, it has been discovered that FAAH actively regulates eCB concentration and creates an “eCB-tone” to preserve the exact shape of the rat germ cells [112]. However, compared to SPC and SPT, 2-AG concentrations were found to be higher in SPG. This was connected to decreased MAGL levels and increased DAGL transcriptional and translational levels in germ cells [110]. Notably, a significantly elevated testicular temperature was linked to restricted germ cell meiosis and higher apoptosis in spermatogonia in TRPV1 mutant animals. In order to protect the germ cells from heat stress, a steady AEA level in the testes for TRPV1 activation may be beneficial [113]. According to a study by De Toni et al. TRPV1 plays a part in mediating thermotaxis in human spermatozoa. The spermatozoa travelling across the temperature range showed an elevated TRPV1 [114]. Fibronectin-induced capacitation in human spermatozoa is also mediated by the TRPV1 receptor [115].

### 3.5. The Endocannabinoid System in Spermatozoa and Its Effect on Spermatozoa Functions

The presence of eCBs in human seminal plasma, sea urchin, amphibian cloacal fluid, boar spermatozoa, bovine sperm, and mouse epididymis clearly shows their harmful impact on spermatozoa’s ability to function correctly [100]. Several studies have linked eCBs with a negative impact on sperm motility in a variety of mammalian species, including humans [91]. These findings have also been reported in knockout models, highlighting the primary function of CB1 signalling in modulating acrosome response, capacitation, sperm motility, and fertilizing competence [116]. This was further buttressed in a study that reported an unfavorable relationship between sperm motility and the concentrations of OEA and AEA in blood serum and seminal fluid [100,116]. The total amount of sperm correlated positively with semen palmytoylethanolamide (PEA), while improved sperm morphology was associated with higher levels of OEA and PEA in the seminal fluid [116].

The various spermatozoa regions exhibit unique expressions of the ECS elements, with FAAH and NAPE-PLD expressed in the mid-piece and post-acrosomal regions [117,118,119]. CB1 is found in the plasma membrane, the acrosomal region, the mid-piece, and at the tip of spermatozoa [120]. A functioning TRPV1 is mainly located in the post-acrosomal zone and the cell’s tail, while CB2 is present in the sperm head plasma membrane [120]. The endocannabinoid membrane transporter (EMT) carries the AEA produced by NAPE-PLD outside spermatozoa [121]. Thus, the eCB produces diverse effects by acting on the CB1 and CB2 receptors, which are widely distributed in the spermatozoa. When triggered, CB1 exacerbates the production of more immobile spermatozoa, whereas when activated, CB2 exacerbates the production of slower spermatozoa [122].

### 3.6. The Endocannabinoid System and Testicular Functions Are Linked

The CB1 may contribute to developing Adult Leydig cells (ALCs) and manifests in the testicular capillary layer before entering germ cells. Cacciola et al. investigated the mRNA and protein expression of CNR1 in rats from postnatal day 7 to 90, during prepubertal testicular development, to gain an understanding [79]. Upon the appearance of round spermatids in the seminiferous epithelium, the expression of cnr1 mRNA peaked at PND7, declined from PND14 to PND31, and then increased at PND35 [79]. Beginning with PND 14, they discovered CNR1 in spindle-shaped interstitial cells, suggesting it plays a part in producing ALCs. After transforming Leydig progenitor cells, the spindle-shaped immature mesenchymal cells develop into ALCs. By PND 28, these progenitor Leydig cells develop into immature Leydig cells [79]. Rat progenitor Leydig cells (PND14) and immature Leydig cells (PND31) were found to be immunopoietic for CNR1 [79]. The immature Leydig cells multiply once at around PND41 before converting into ALCs between PND28 and PND56. They found that CNR1 levels were considerably lower than PND35 at this point. The CNR1 was much greater at PND35, and from PND 60 to 90, there was a comparable upward trend in CNR1. They proposed that age and a spike in ALC numbers may be related to changes in CNR1 expression.

Additionally, Negative staining for CNR1 was found on immature mitotic Leydig cells, while positive staining for CNR1 was observed on immature non-mitotic Leydig cells in the same cohort. They proposed that CNR1 plays a detrimental role in the proliferation of adult Leydig cells (ALCs) at this stage [79]. Thereby indicating that the CB1 receptor has a detrimental role in ALC proliferation at this point, which is also linked to reduced basal testosterone output in CB1 KO testes [79,119]. However, contradictory reports suggest CB1 to be positively associated with differentiation activities that supervise the ontogenesis of Leydig cells [123].

A distinct EC signalling pattern was identified in various human testicular cells, spanning from germ cell development stages, like post-meiotic germ cells, to somatic cells [76]. The ECS is therefore directly involved in the regulation of human testicular physiology, encompassing spermatogenesis and Leydig cell function [76].

Other ECS elements have also been reported in rodent testes, mainly at the transcriptional level [124]. AEA synthesis has been reported in rat testis, and it was strongly expressed in Leydig cells and Sertoli cells, the latter of which had the full capacity to transport, degrade, and bind AEA through CB2. Higher levels of FAAH counteract this eCB’s pro-apoptotic action on Sertoli cells [125]. The presence of ECS receptors and eCBs in the testis suggests their role in spermatogenesis and steroidogenesis despite limited knowledge about eCBs’ physiological effects on the testis [126].

### 3.7. The Relationship Between the Endocannabinoid System and Male Sex Hormones

It is evident that the ECS is crucial to spermatogenesis and, consequently, male reproduction. Furthermore, the interdependent relationships between it, testosterone, and estrogen demonstrate the ECS’s key involvement in hormone control. The development of the blood–testis barrier, spermatogenesis, spermiogenesis, and the ejection of mature sperm all depend on testosterone [74,92]. Infertility and unsuccessful spermatogenesis are linked to testosterone insufficiency or androgen receptor absence.

Sertoli cells are the primary target of testosterone signalling, while Leydig cells are the location of testosterone manufacture. Numerous research has demonstrated the detrimental effects of cannabis on testosterone synthesis, as was previously mentioned [125]. Male sexual behaviour changes due to lower testosterone levels, which might result in infertility [127]. According to Wenger et al., in wild-type mice, the elevated AEA levels cause the LH and testosterone levels to decrease; however, in CB1KO mice, the hormone levels stay unchanged [128]. Likewise, THC treatment decreased testosterone release in cultured Leydig cells [128]. However, Tarumi and Shinohara reported that olfactory exposure to β-CBP elevated testosterone levels in vitro [129].

There is also evidence on the importance of estrogens and their receptors (ER) in male reproduction, in addition to testosterone. Both ER α and ER β are expressed in the mammalian testis, which is how estrogens work and control male reproduction [130]. Similarly, specific testicular cells have been shown to produce and activate aromatase, an enzyme that converts androgens to estrogens irreversibly [130,131].

The link between estrogens and ECS is demonstrated by the discovery that FAAH is a direct gene controlled by 17β-estradiol (E2) in mouse Sertoli cells. Only ERβ is expressed by mouse Sertoli cells [132]. The binding of ERβ to ERE sites in the FAAH promoter via a direct ligand-dependent mechanism leads to epigenetic changes affecting transcription. The study noted a heightened interaction between E2-mediated ERβ and ERE2/3 promoter sites [74]. FSH further controls the production and activity of FAAH in Sertoli cells by activating the protein kinase A (PKA)/aromatase pathway [132]. While the aromatase mechanism includes inducing FAAH transcription via the estrogens as aromatase transforms testosterone to estrogens, the PKA pathway requires inducing phosphorylation of the proteins that trigger FAAH [125].

**Figure 13:**
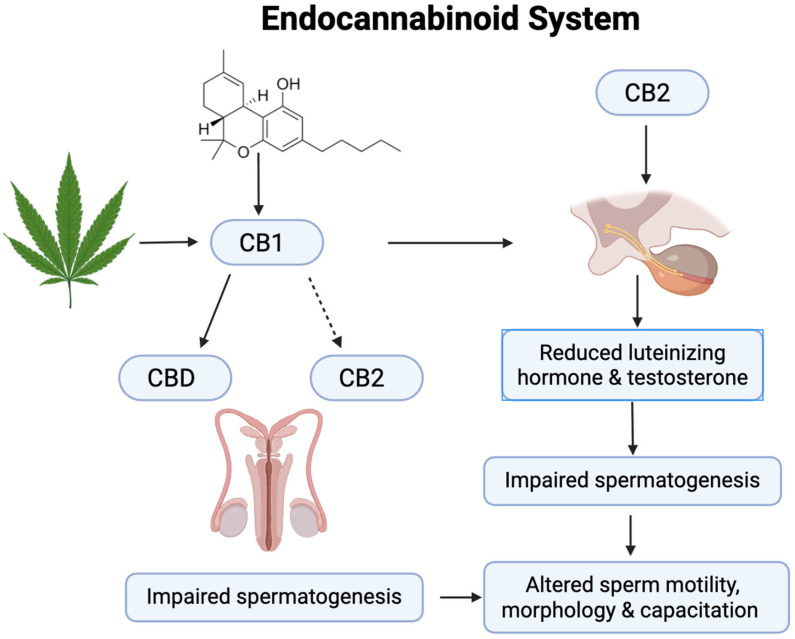
A schematic presentation of ECS and its involvement in sperm function and testosterone regulation.

A summary of ECS and its involvement in sperm function and testosterone regulation is depicted in Figure 13.

### 3.8. Effect of Cannabis on the Cation Channel of Sperm (CatSper)

The cation channel of sperm (CatSper) is the principal Ca^2+^ channel in the sperm plasma membrane, and it specifically localizes to the flagellum; it plays a crucial role in modulating the downstream processes that are involved in fertilization [133,134,135]. THC and CBD have been reported to modulate the sperm-specific Ca^2+^ channel CatSper, by suppressing the activation of the channel by progesterone (P4) and prostaglandin E1 (PGE1) [133,136]. Thus, indicating them as genuine CatSper inhibitors [133]. The inhibition of CatSper suppresses the intracellular Ca^2+^ concentration, leading to impaired major sperm functions such as capacitation, hyperactivation, and acrosome reaction [137,138].

### 3.9. Cannabis and Sperm Epigenetics

Fetal development is heavily reliant on sperm, which utilize epigenetic mechanisms to regulate it, and any disruptions to these mechanisms can result in altered epigenetic markers being passed down from father to child [139]. Changes in sperm epigenetics may also lead to abnormalities in semen quality and reduced fertility, which can impede fertilization, lower conception rates, and negatively impact early embryonic development [140,141].

The use of cannabis has been associated with altered sperm epigenetic markers, which may alter the expression of genes in the fetus [139,142]. Cannabis and Δ^9^-THC have an impact on the sperm epigenome, specifically affecting DNA methylation, which can impact developmental genes like DLGAP2 and potentially affect the health of offspring [140].

In rodents, exposure to paternal Δ^9^-THC before conception results in cholinergic synaptic dysfunction, unusual motor activity, impaired cognitive abilities, or lasting neurobehavioural effects in their offspring [143,144]. Watson et al. demonstrated that exposure to Δ^9^-THC in adolescent rats led to cross-generational impacts on the DNA methylation status in the nucleus accumbens (NAc) [145]. DNA methylation changes in the DLGAP2 gene were found in the NAc of the offspring of rats and were associated with the potential development of an autism-like condition in rats born to fathers exposed to Δ^9^-THC before conception [146]. Shi et al. demonstrated that male mice exposed to cannabis vapor showed a decrease in both sperm count and/or motility among F0 and F1 males, with concomitant increased DNA damage [147].

### 3.10. Therapeutic Potentials of Phytocannabinoids vs. Whole Plant Toxicity

While there is still much to understand, current evidence suggests that cannabinoids may have both positive and negative effects on male fertility. These effects are primarily related to their impact on the reproductive system, including sperm quality, hormone levels, and sexual function.

Costa et al. studied the psychoactive compound of *C. sativa*, THC, which reduces the turnover of human trophoblast cells [148]. However, its effects on these cells remain unclear, particularly during syncytialization and regarding the survival or mortality of primary human cytotrophoblasts (CTs) and syncytiotrophoblasts (STs). Although the effects on STs were more pronounced, the study revealed that THC exerted a dual impact on both trophoblast phenotypes. THC enhanced MTT metabolism at low levels, whereas at higher doses, it reduced cell survival. Furthermore, THC decreased the production of oxidative and nitrative stress, as well as the oxidized form of glutathione, while increasing its reduced form. This suggests that THC may have an antioxidant effect that helps prevent ST cell death. Additionally, THC inhibited the development of CTs into STs through a cannabinoid receptor-dependent mechanism, reducing the expression of biochemical and morphological markers of syncytialization. These findings suggest that THC interferes with trophoblast turnover, thereby inhibiting both trophoblast cell death and differentiation. They also provide insight into the biological processes that may contribute to pregnancy complications in women using cannabis-derived medications during pregnancy [148].

Erukainure et al. reported the protective potentials of *C. sativa* sequential extracts on oxidative-mediated testicular injury in isolated rats’ testis, ex vivo [17]. Testicular tissues were co-incubated with *C. sativa* extracts and the pro-oxidant for 30 min. Biochemical analyses revealed elevated antioxidant enzyme activities, mitigation of purinergic and cholinergic dysfunctions, and improved glucose metabolism [17].

Taiwo et al. orally exposed pre-puberty male rats to CBC for 21 days [149]. They observed elevated activities of arginase and phosphodiesterase-5, with concomitant depleted levels of nitric oxide (NO) and calcium in the penile tissues of CBC-exposed rats. Spermatoxic activities and suppressed steroidogenesis, with decreased serum levels of testosterone, FSH, and LH, indicated an inhibitory effect of CBC on spermatogenesis. They concluded that CBC mediates infertility in pre-puberty males by stimulating erectile dysfunction, inhibiting steroidogenesis, and downregulating genes linked with male fertility [149].

Espinosa-Ahedo et al. investigated the potential of β-CBP to protect against cadmium chloride-induced male reproductive toxicity [150]. Male rats were pre-administered β-CBP (20, 200, and 400 mg/kg bodyweight), before being exposed to a single dose of 3 mg/kg CdCl_2_. The rats were further administered β-CBP for 6 days. β-CBP improved sperm concentration, mobility, viability, and morphology. Furthermore, β-CBP caused a reduction in micronuclei in the spermatids, while mitigating CdCl_2_-induced testicular oxidative stress and restoring testicular morphology [150].

López-Cardona et al. investigated the effects of long-term THC use on the reproductive organs, sperm, and in vitro fertilization in male mice [151]. The mice were randomly assigned to two groups: one group received a daily dose of 10 mg/kg body weight of THC for 30 days, while the other received a vehicle. In the THC-treated mice, Cnr1 mRNA levels were significantly lower in the brain compared to the control mice, although Cnr1 expression remained unchanged in the testes. Histological evaluation and measurements of testicular and epididymal weights revealed no significant differences between the groups. Additionally, sperm concentration and motility were unaffected. Examination of the three CpG regions in the Cnn1 gene showed no changes in methylation due to long-term THC use, either in the brain or in IVF embryos. Furthermore, no differences were observed in the IVF success rates when sperm from THC-exposed and control mice were used. This study contradicts the notion that THC use adversely affects male reproductive health [151].

Whan et al. explored the effects of Δ^9^-THC on human sperm function in vitro [91]. The study involved computer-assisted semen analysis, acrosome reaction assessment using fluorescein isothiocyanate-labeled peanut agglutinin staining, an assisted reproductive technology unit, and seventy-eight male patients to evaluate sperm motility following THC exposure. Sperm were separated into two fractions using density centrifugation: 90% (the highest fertilizing potential, used in assisted conception) and 45% (a lower-quality subpopulation). These fractions were then incubated with THC at therapeutic (0.032 µM) and recreational (0.32 and 4.8 µM) blood concentrations for three hours at 37 °C. Sperm motility, as well as artificial and spontaneous acrosome responses, were assessed. There was a dose-dependent reduction in progressive motility, with a decrease range of 2 to 21%. The 45% fraction showed a more significant decline in motility, with a reduction of 28 and 56% at 0.032 and 4.8 µM, respectively. Additionally, both fractions exhibited a decrease in average path velocity and straight-line velocity, with a 10% reduction in the 90% fraction. The 90% fraction showed a decline in spontaneous acrosome response by 17 and 35% at 0.032 and 4.8 µM, respectively. The 45% fraction showed a decrease ranging from 17 to 35% (*p* < 0.001). When A23187 was used to induce the acrosome response artificially in the 90% fraction, THC (4.8 µM) resulted in a 57% inhibition. Based on these findings, the study suggests that recreational use of THC could negatively affect male fertility [91].

Abbas et al. studied the effects of β-caryophyllene on endometriosis, fertility, and reproduction in adult female rats [152]. Autologous endometrial fragments were implanted into the peritoneal cavity of the rats. After four weeks, the growth of the endometriotic implants was measured. Following 21 days of treatment with either a vehicle (control) or β-caryophyllene (at doses of 10 mg/kg or 30 mg/kg), the development of the endometriotic implants was reassessed. In fertility experiments, female rats treated with either vehicle or β-caryophyllene were mated, and various reproductive parameters were assessed, including the quantity and quality of implants, the number of corpora lutea, pregnancy duration, and litter outcomes. β-caryophyllene (10 mg/kg) reduced the development of endometriotic implants by 52.5% compared to controls. Additionally, β-caryophyllene induced apoptosis in the blood vessel endothelial cells and the cyst’s luminal epithelium. Ultrastructural analysis further showed that control and β-caryophyllene-treated rat cysts contained active mast cells and eosinophils. In the fertility experiments, no significant differences were found between the control and β-caryophyllene-treated groups for any of the parameters evaluated. They concluded that β-caryophyllene therapy may offer a promising new, non-toxic treatment alternative for the treatment of endometriosis [152].

Sam investigated the effects of the psychoactive substances Δ^9^-THC and Δ^8^-THC on sperm mitochondrial O_2_ consumption (respiration) [87]. Forty-one men visited the andrology lab for fertility assessment. Sperm respiration was measured using a phosphorescence analyzer, which tracks the O_2_ concentration in sperm suspensions over time. When Δ^9^-THC or Δ^8^-THC was administered to washed sperm, the rate at which it respirated immediately decreased. Δ^9^-THC was the more potent of the two substances, with a concentration-dependent inhibitory activity. Adding either Δ^9^-THC or Δ^8^-THC to neat semen significantly reduced respiratory activity, suggesting the presence of protective factors in seminal plasma. Both substances also inhibited mitochondrial respiration in isolated mitochondria, indicating that direct mitochondrial damage is likely the primary mechanism of action. As the two main active cannabinoids in marijuana, Δ^9^-THC and Δ^8^-THC are strong inhibitors of human sperm mitochondrial O_2_ consumption. These findings emphasize the negative impact of these compounds on male fertility [87].

Ricci et al. reported the endocannabinoid’s physiological role in the male reproductive system [153]. They demonstrated how endocannabinoids influence the epididymis using CB1 knock-out (CB1KO) and wild-type (WT) mice. In the study, sperm motility, as measured by the proportion of motile spermatozoa (SPZ), was inhibited by CB1. Specifically, in WT mice, the proportion of motile SPZ was significantly lower in the caput compared to the cauda of the epididymis, as expected. However, in CB1KO mice, motility was notably higher in the caput than in the cauda [153].

Alagbonsi and Olayaki evaluated the effects of melatonin and/or THC on the movement and motility of capacitating rat sperm, in vitro [154]. Rat semen was randomly divided into nine treatment groups (*n* = 5). Groups 1–4 were treated with AM-630 (1 mM), THC (1 mM), SR141716 (1 mM), and a placebo, respectively. In groups 5–7, THC was administered five minutes after pretreatments with SR141716, AM-630, or their combination. Group 9 received THC and melatonin, while Group 8 received melatonin (5 mM). Inhibition of cannabinoid receptors (CB1 or CB2) partially reversed the THC-induced reduction in sperm motility and kinematics, while blocking both CBs eliminated the effect. Interestingly, when administered alone, melatonin improved the progressive motility and kinematics of rat sperm and counteracted the THC-induced decline in these parameters by 42%. Melatonin and/or cannabis treatments had a lesser impact on the acceleration ratios of hyper-activated motility in capacitated sperm than sperm velocities, lateral head amplitude, and beat/cross frequency. They concluded that melatonin reduces THC -induced spermatotoxic effects, via modulation of CB1 and CB2 receptors [154].

Carvalho demonstrated that prolonged exposure to CBD impairs male mice’s fertility and sexual behaviour [155]. Twenty-one-day-old male Swiss mice were orally administered CBD at oral doses of 15 or 30 mg/kg daily for 34 days. The CBD-treated animals showed no significant changes in weight gain or circulating progesterone levels. Sexual behaviour studies revealed that at 15 mg/kg, CBD exhibited fewer mounts and ejaculations, along with the delayed onset of the first mount and intromission. At 30 mg/kg, CBD decreased the reproductive rate and the number of litters by 30 and 23%, respectively. These findings suggest that extended CBD exposure negatively affects the reproductive function of male Swiss mice [155].

Almada et al. investigated the effects of THC and CBD on E2 metabolism/signalling, ESC proliferation, and differentiation [156]. They discovered that THC does not impair the differentiation of ESCs, but CBD does. They further demonstrated that CBD inhibits the rise in E2 levels, and CYP19A1 gene transcript levels are seen in differentiating ESCs. They further reported that CBD had anti-aromatase activities. Overall, they pointed to a new way that CBD affects human endometrial differentiation, which could result in infertility issues [156].

Alagbonsi and Olayaki investigated the role of oxidative stress in spermatotoxicity associated with *C. sativa* [157]. They examined the potential benefits of combining melatonin and vitamin C, as opposed to administering them separately [157]. *Cannabis sativa* reduced sperm parameters, TAC, and the ROS-TAC score, and increased ROS levels in male albino rats. Co-administration of melatonin or vitamin C with *C. sativa* exacerbated oxidative stress and spermatotoxicity. Thereby indicating that *C. sativa* induces oxidative stress and spermatotoxicity, which contributes to male infertility [157].

Park et al. reported that THC and other cannabinoids do not have direct estrogenic effects [158]. However, there is an interaction between marijuana smoking and estrogen receptors [158]. The estrogenic effects are primarily attributed to phytoestrogens in *C. sativa*, such as apigenin, an estrogenic flavonoid that retains its pharmacological activity in marijuana smoke [158]. These phytoestrogens and terpenes present in marijuana can be inhaled, volatilized, and cross the blood-brain barrier, potentially modifying the effects of THC [158]. Long-term marijuana use is also associated with reduced plasma testosterone and serum LH levels in men compared to pre-smoking baseline levels or hormone levels in non-smoking controls [21,78,159].

## 4. Conclusions

In conclusion, this review presented the effect of *Cannabis* on male fertility. Cannabinoids, particularly THC, appear to have a generally negative impact on male fertility, affecting sperm quality, hormone levels, and sexual function. However, the extent of this impact can vary depending on the frequency and amount of use. CBD shows potential for some positive effects, but more research is needed to understand its role in male reproductive health fully. For men concerned about infertility, it may be advisable to limit or avoid cannabis use, especially in the context of attempting to conceive. However, future studies may provide more insights into the potential therapeutic use of cannabinoids for fertility. Overall, the ECS plays a complex and important role in male fertility. Once the molecular events regulated by the ECS in the testis are fully understood, it will be possible to more precisely define the protective role of the ECS in maintaining proper spermatogenesis and the development of mature, fertilizing sperm. Exposure to exogenous cannabinoids may disrupt this system, potentially affecting male fertility by altering the physiological function of the ECS in male reproduction. However, more research is needed to fully understand its impact and potential therapeutic implications. Future studies should also focus on addressing the dose discrepancy of cannabis and its cannabinoids to ensure usage safety, maximize therapeutic effectiveness, and establish consistent product quality.

## Data Availability

All data are presented in the article.
